# Supplementary material for: RNAi-Mediated Screen of Primary AML Cells Nominates MDM4 as a Therapeutic Target in NK-AML with DNMT3A Mutations
Source: Cells. 2022 Mar 2;11(5):854. doi: 10.3390/cells11050854 (PMC8909053; doi:10.3390/cells11050854)
Supplement: Supplementary file 1 [file cells-11-00854-s001.zip › cells-1544439 supplementary new/supplementary.pdf]

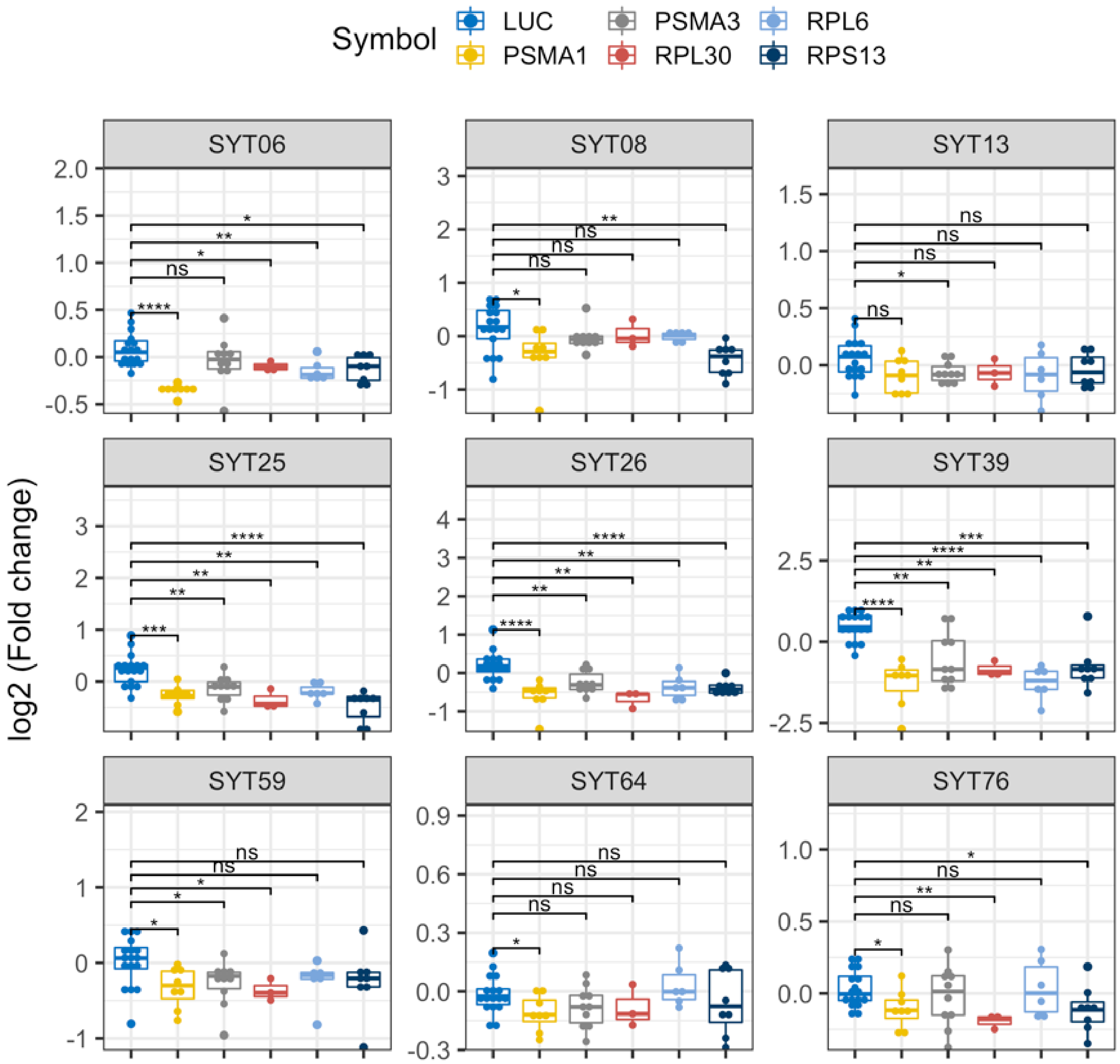

Figure S 1. Boxplots showing the median fold change of the positive control shRNAs in screens performed in 9 primary AML lines. The fold changes were compared to the median fold change of the non-targeting control shRNA against luciferase (LUC) using the Wilcoxon rank-sum test. The p-value was adjusted for multiple testing using the false discovery rate methof by Benjamini and Hochberg (*: p ≤ 0.05; **: p ≤ 0.01; ***: p ≤ 0.001; ****: p ≤ 0.0001).

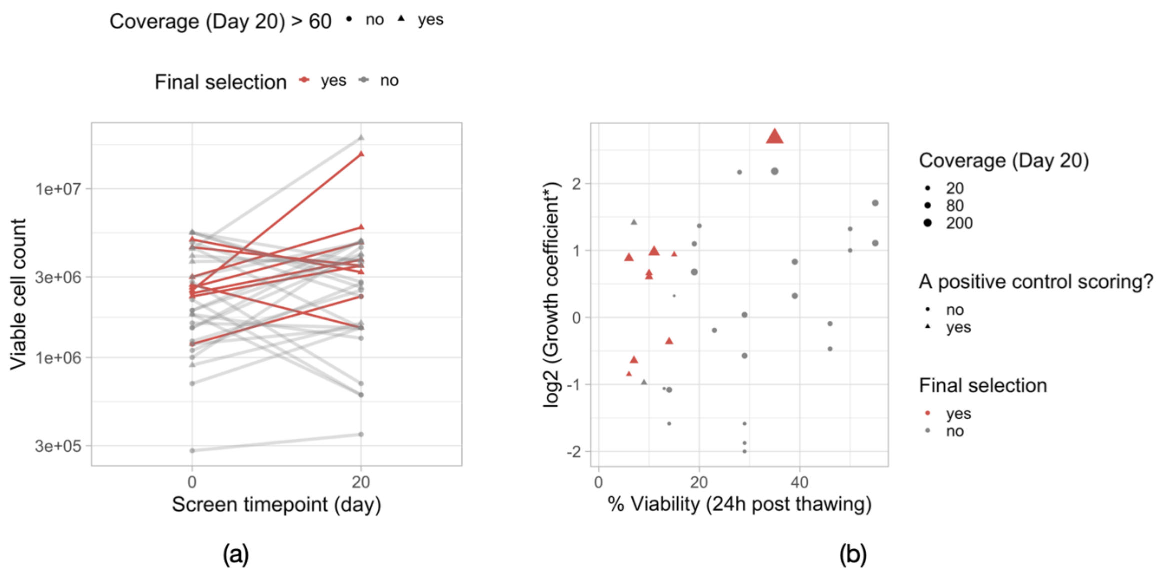

Figure S 2. A summary of the primary cells' viability and growth data, as well as library coverage and the positive control behavior. (a) A slope chart showing the number of viable primary AML cells at the beginning (Day 0) and at the end (Day 20) of the functional screen period. The samples with the coverage of >60x as well as the 9 samples selected for the pooled analysis (final selection) are marked. (b) A correlation plot shwoing the relationship between the initial viability of the primary AML cells, their growth coefficient, the coverage they achieved by the end of the screening period and the positive control behavior. The 9 samples selected for the pooled analysis are marked. * - the growth coefficient is the ratio of viable cells on Day 20 and Day 0.

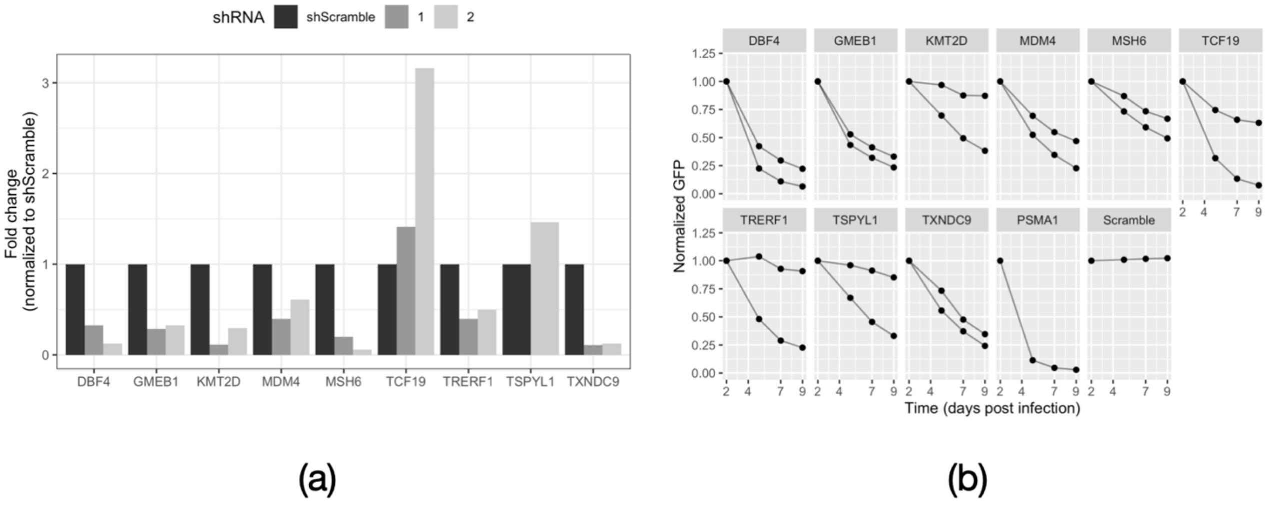

Figure S 3. (a) Analysis of the mRNA expression levels in OCI-AML3 cells expressing shRNAs against candidate genes nominated in the primary AML screen. N=1 (b) The growth dynamic of OCI-AML3 cells expressing the two top-performing? shRNAs against the candidates nominated in the primary AML screen). N=1. PSMA1 is a positive control gene, Scramble is a non-targeting control (shScramble).

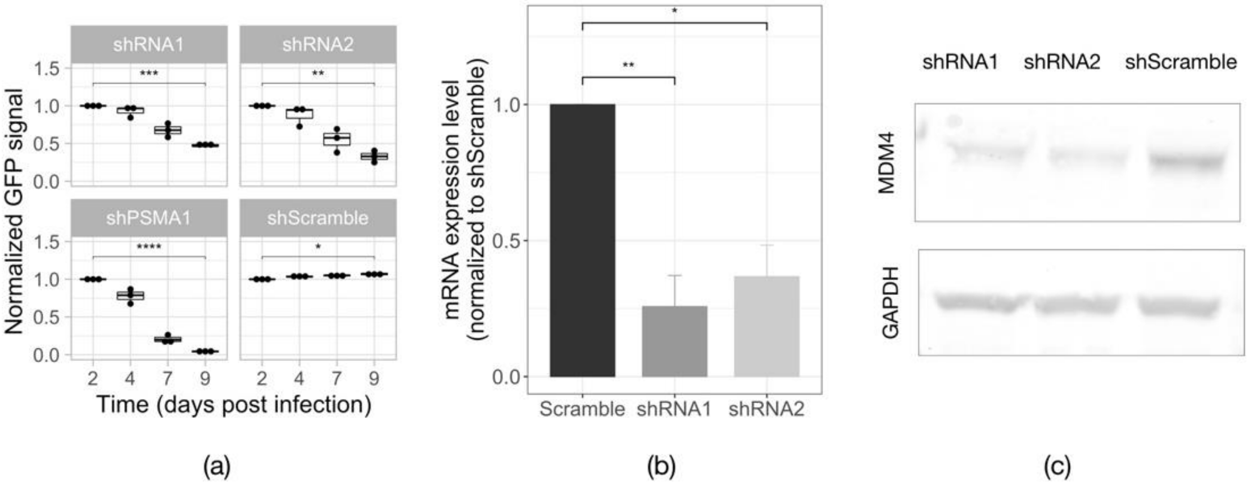

Figure S 4. Validation of the screen phenotype and the on-target activity of the selected shRNAs against MDM4. (a) Validation of the screen phenotype with a GFP reporter assay. OCI-AML3 cells carrying the shRNA are marked by GFP expression. The GFP signal was measured over time by flow cytometry as a proxy for the growth dynamic of the shRNA-expressing cells (N=3). The data is normalized to day 2 post infection. shPSMA1 – positive control shRNA, shScramble – non-targeting shRNA. The p-values were calculated using the student's paired t-test (*: $p \leq 0.05$; **: $p \leq 0.01$; ***: $p \leq 0.001$; ****: $p \leq 0.0001$). (b) Validation of the on-target activity of the MDM4 shRNAs in OCI-AML3 cells by qPCR (N=3). TBP was used as the housekeeping control gene. The values are normalized to the non-targeting control shRNA (Scramble). (c) Validation of the on-target activity of the MDM4 shRNAs on the protein level by western blot in HeLa cells (N=2). A representative blot is shown. GAPDH was used as the loading control.

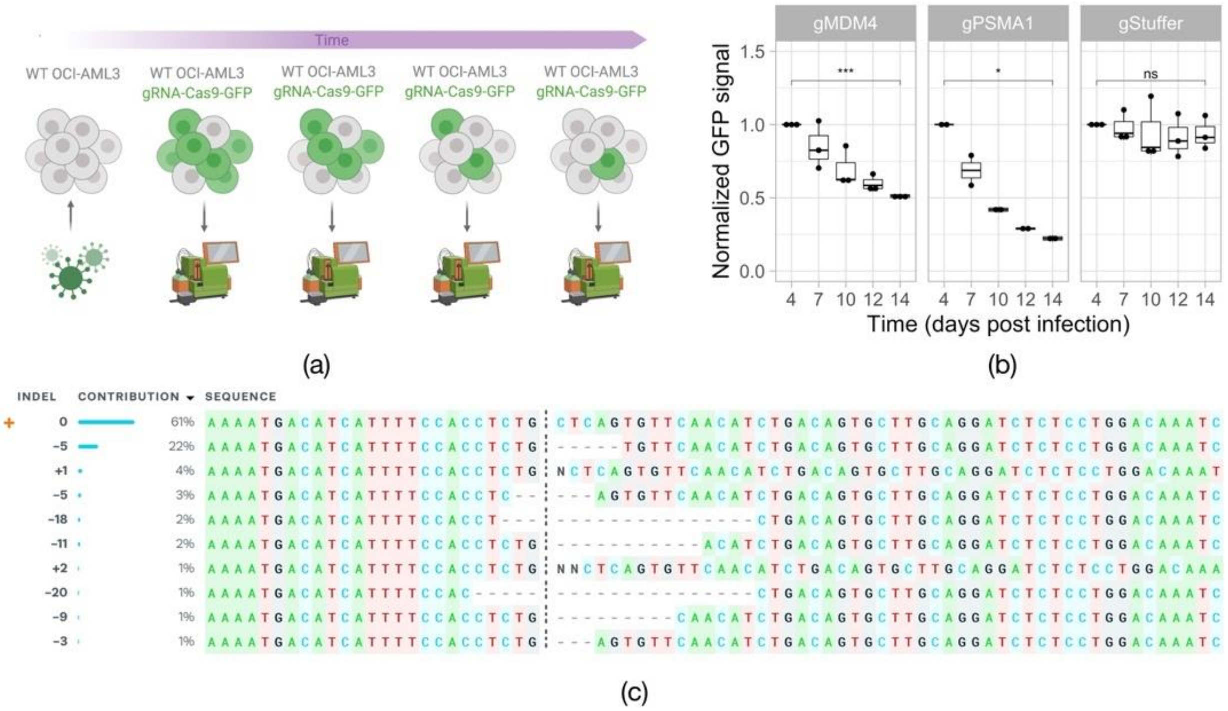

Figure S 5. The summary of the CRISPR/Cas9-mediated knock out of MDM4 in OCI-AML3 cells. (a) The pipeline of the GFP reporter assay: OCI-AML3 cells were infected with a lentivirus containing the gRNA-Cas9-T2A-GFP construct. The cells that received the construct were cultured together with the wild type cells for 2 weeks. The GFP signal was measured over time by flow cytometry as a proxy for the growth dynamic of the cells expressing the gRNA and the Cas9 protein. Created with BioRender.com (b) Quantification of the flow cytometry data obtained from the GFP reporter assay described in (a). The data is normalized to day 4 post infection. gPSMA1 – positive control gRNA; gStuffer – Cas9-T2A-GFP only (no gRNA) control. The p-values were calculated using the Student's paired t-test (*: $p \leq 0.05$; **: $p \leq 0.01$; ***: $p \leq 0.001$; ****: $p \leq 0.0001$; ns – not significant). N=3. (c) Analysis of the indel sequences generated upon expression of Cas9 together with MDM4-gRNA in HEK293T cells. Performed with ICE CRISPR Analysis Tool from Synthego.

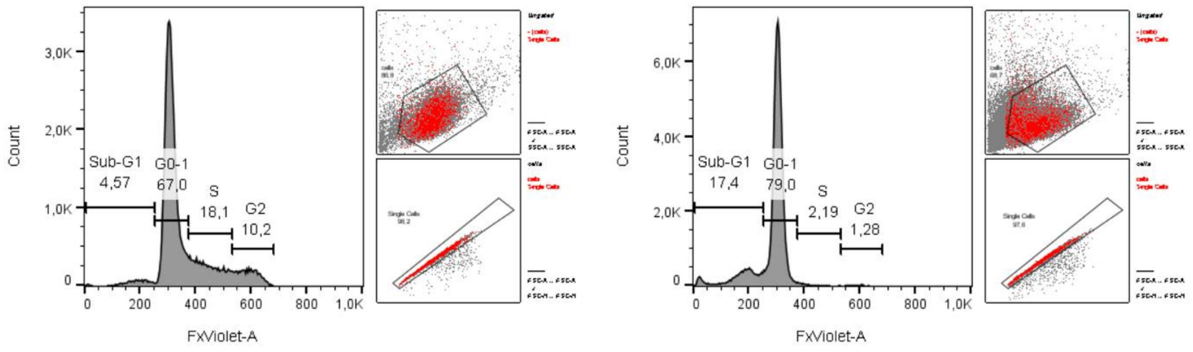

Figure S 6. Cell cylce analysis of the primary AML sample SYT1788. The left panel shows the DMSO-treated sample, the right panel shows the sample treated with 5 μM ALRN-6924. Both samples were analysed 24h after the start of the treatment. The cell cycle phases are annotated, the apoptotic cells fall into the Sub-G1 gate. The smaller panels depict the backgating.

Table S1: PCR primers

#	Name	Sequence	Ta
1	U6-seq-F	GAGGGCCTATTTCCCATGATT	56
2	shRNA-seq-R	GGCAGCGCTCGCCGTGAGGA	65
3	gRNA-seq-R	GCTGTTTCCAGCATAGCTCT	57
4	F2	TCGGATTCGCACCAGCACGCTA	66
5	GexSeqS-R	AGAGGTTCAGAGTTCTACAGTCCGAA	61
6	P7-tail-F	GTGACTGGAGTTCAGACGTGTGCTCTTCCGATCTTCGGATTCGC ACCAGCACGCTA	66
7	P5-tail-R	ACACTCTTTCCCTACACGACGCTCTTCCGATCTAGAGGTTCAGA GTTCTACAGTCCGAA	61

Table S2: Oligos for cloning of shRNAs and gRNAs

Name	Sequence
DNMT3A-R882H-gRNA top	CACCGTGAGCCACTTGGCGAGGCAG
DNMT3A-R882H-gRNA bottom	AAACCTGCCTCGCCAAGTGGCTCAC
MDM4-shRNA1 top	ACCGGGTTCACTGTTAAAGAGGTTATGTTAATATTCATAGCA TGACCTCTTTAACAGTGAACTTTT
MDM4-shRNA1 bottom	CGAAAAAAGTTCACTGTTAAAGAGGTCATGCTATGAATATT AACATAACCTCTTTAACAGTGAACCCGGT
MDM4-shRNA2 top	ACCGGTGCCTACCTCAGAGTATAAATGTTAATATTCATAGCA TTTATGCTCTGAGGTAGGCATTTT
MDM4-shRNA2 bottom	CGAAAAAATGCCTACCTCAGAGCATAAATGCTATG AATATTAACATTTATACTCTGAGGTAGGCACCGGT
PSMA1-shRNA top	ACCGGGCAATGGAAGCTGTTAAATAAGTTAATATTCATAGC TTGTTTAACAGCTTCCATTGCTTTT
PSMA1-shRNA bottom	CGAAAAAAGCAATGGAAGCTGTTAAACAAGCTATGAATATT AACTTATTTAACAGCTTCCATTGC
Scramble shRNA top	ACCGGCAACAAGATGAAGAGCACCAAGTTAATATTCATAGC TTGGTGCTCTTCATCTTGTTGTTTT
Scramble shRNA bottom	CGAAAAAACAACAAGATGAAGAGCACCAAGCTATGAATAT TAACTTGGTGCTCTTCATCTTGTTG
MDM4-gRNA top	CACCGAGATGTTGAACACTGAGCAG
MDM4-gRNA bottom	AAACCTGCTCAGTGTTCAACATCTC
PSMA1-gRNA top	CACCGTACCAATATGGTTGTCAACA
PSMA1-gRNA bottom	AAACTGTTGACAACCATATTGGTAC

Table S3: qPCR primers

#	Name	Sequence
1	MDM2-F	GCAGTGAATCTACAGGGACGC
2	MDM2-R	ATCCTGATCCAACCAATCACC
3	MDM4-F	TGAACATTTCACCTTGCGCACCTG
4	MDM4-R	CAACATCTGACAGTGCTTGCAGGA
5	P21-F	GGCAGACCAGCATGACAGAT
6	P21-R	GGATTAGGGCTTCCTCTTGG
7	TBP-F	AGGTTAGAAGGCCTTGTGCTC
8	TBP-R	GGAGAACAATTCTGGGTTTGATCA
