# Supplementary figures and images for: RNAi-Mediated Screen of Primary AML Cells Nominates MDM4 as a Therapeutic Target in NK-AML with DNMT3A Mutations
Source: Cells. 2022 Mar 2;11(5):854. doi: 10.3390/cells11050854 (PMC8909053; doi:10.3390/cells11050854)

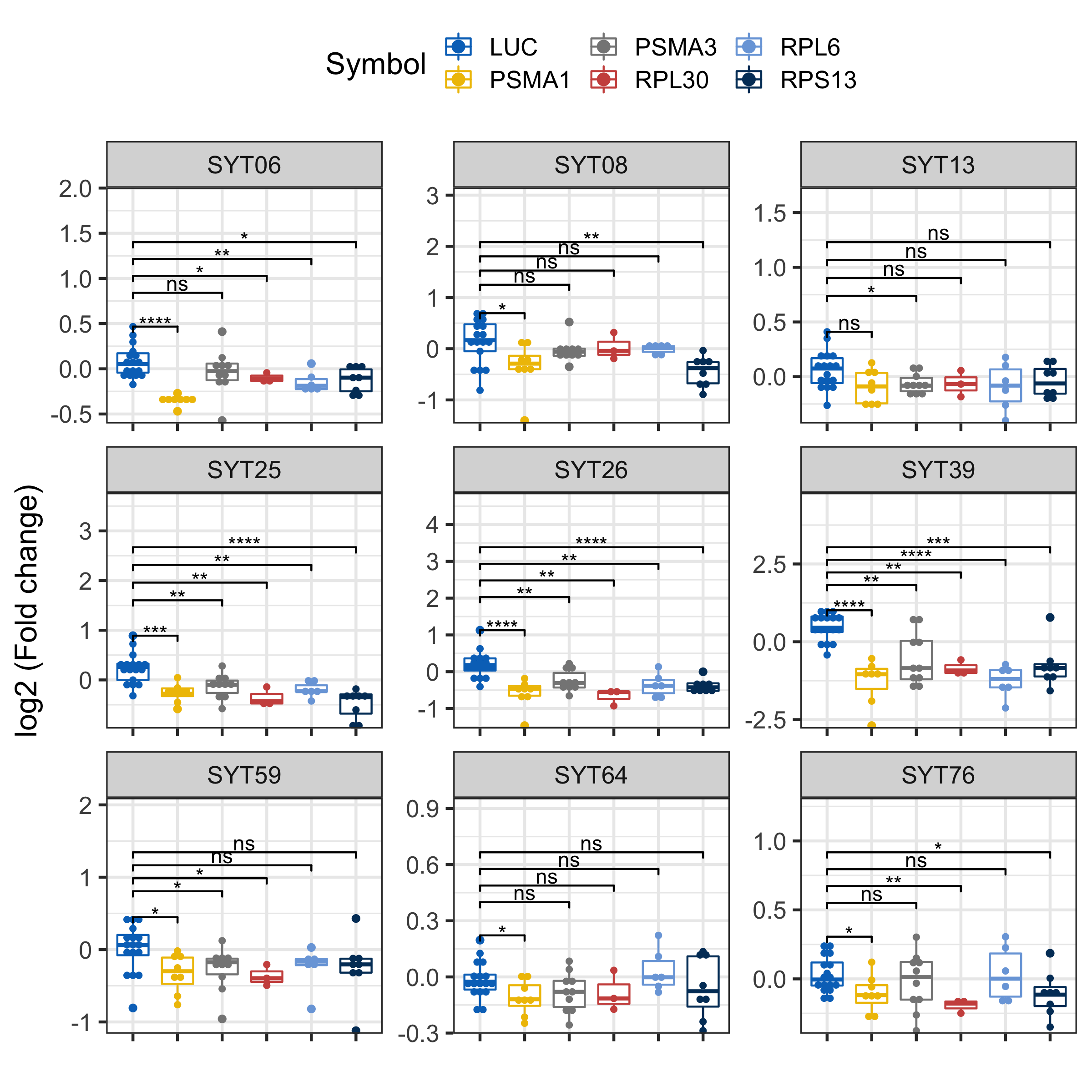

Supplement: Supplementary file 1 [file cells-11-00854-s001.zip › cells-1544439 supplementary new/figureS1.png]

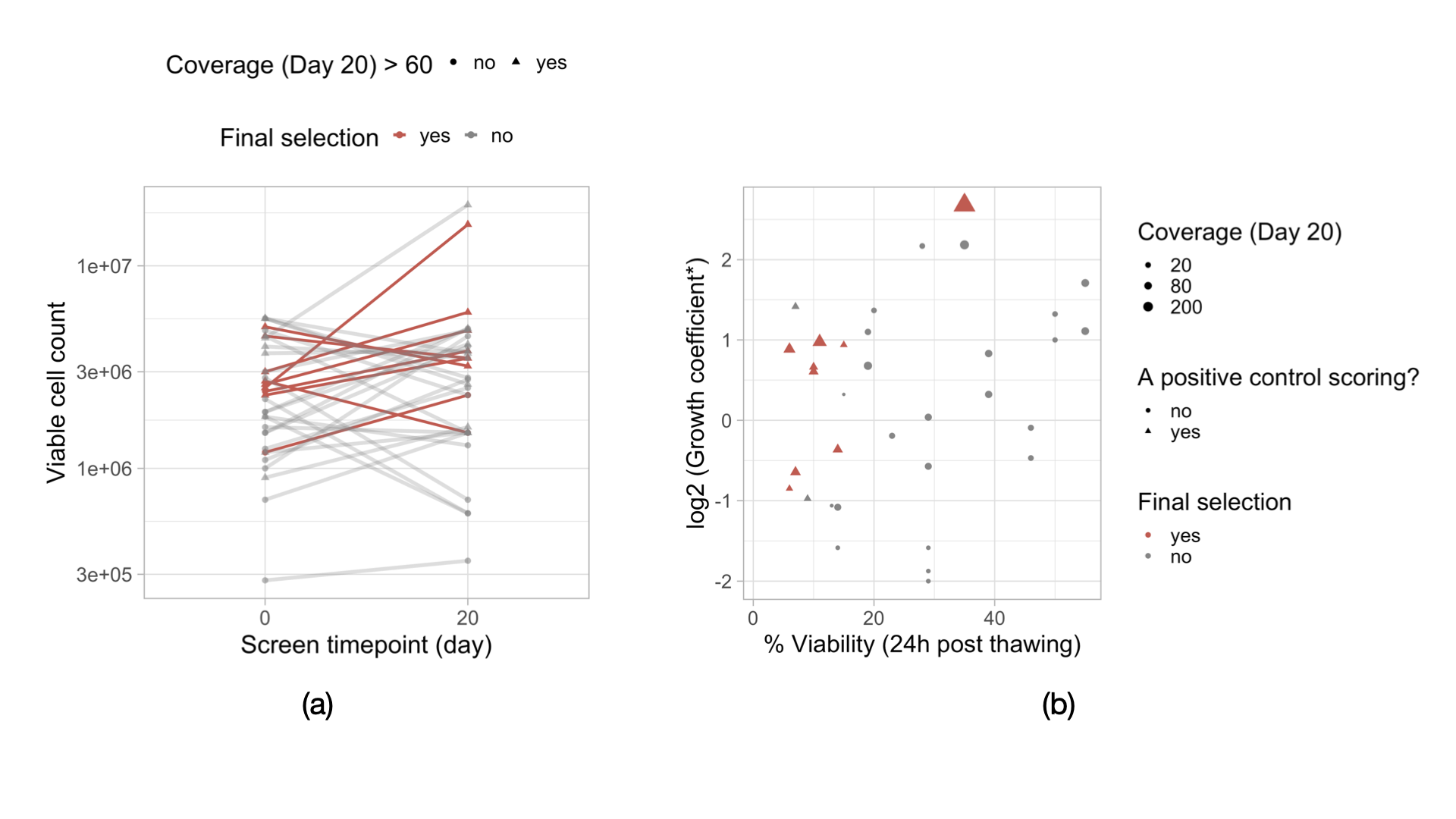

Supplement: Supplementary file 1 [file cells-11-00854-s001.zip › cells-1544439 supplementary new/figureS2_220202.png]

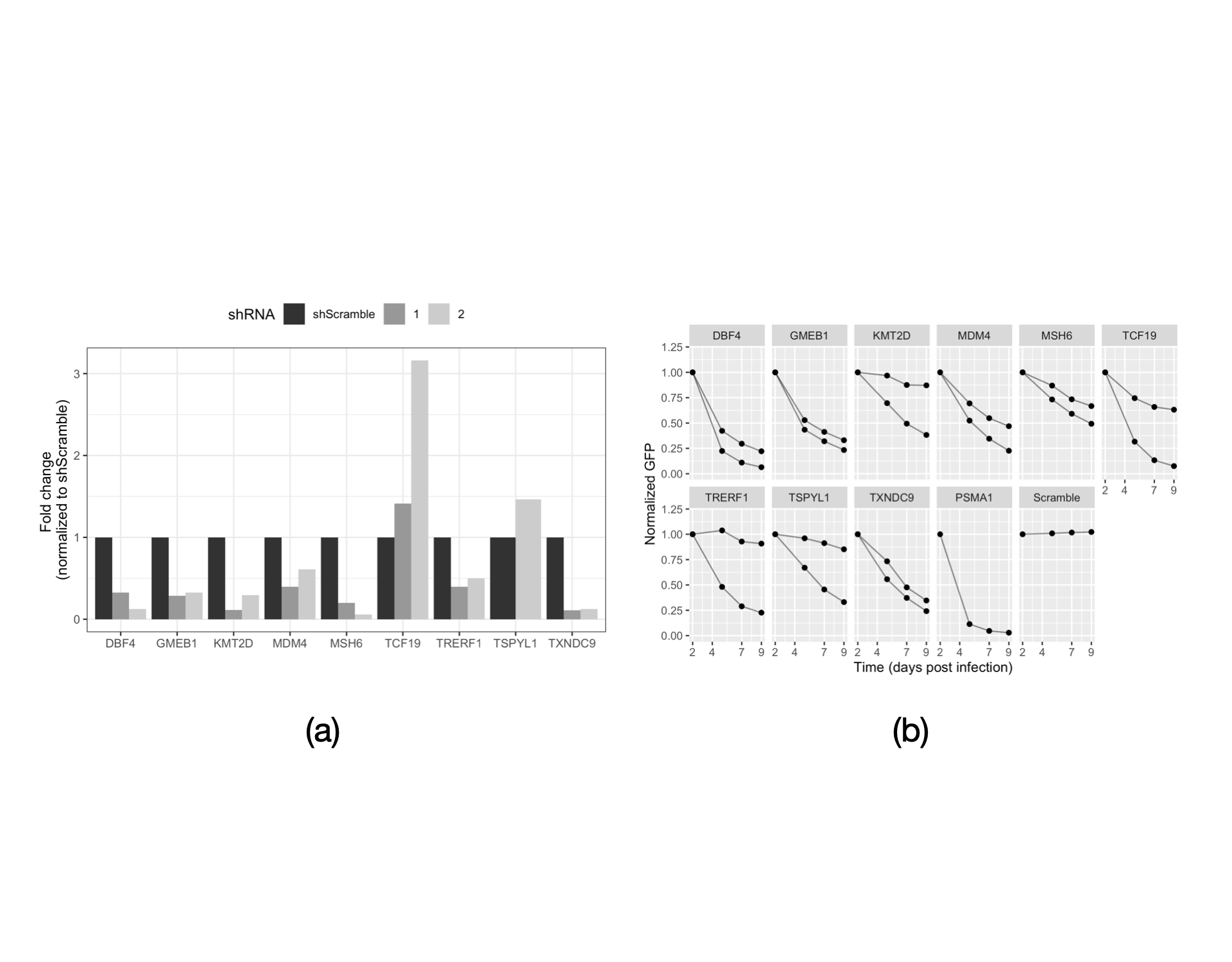

Supplement: Supplementary file 1 [file cells-11-00854-s001.zip › cells-1544439 supplementary new/figureS3_211222.png]

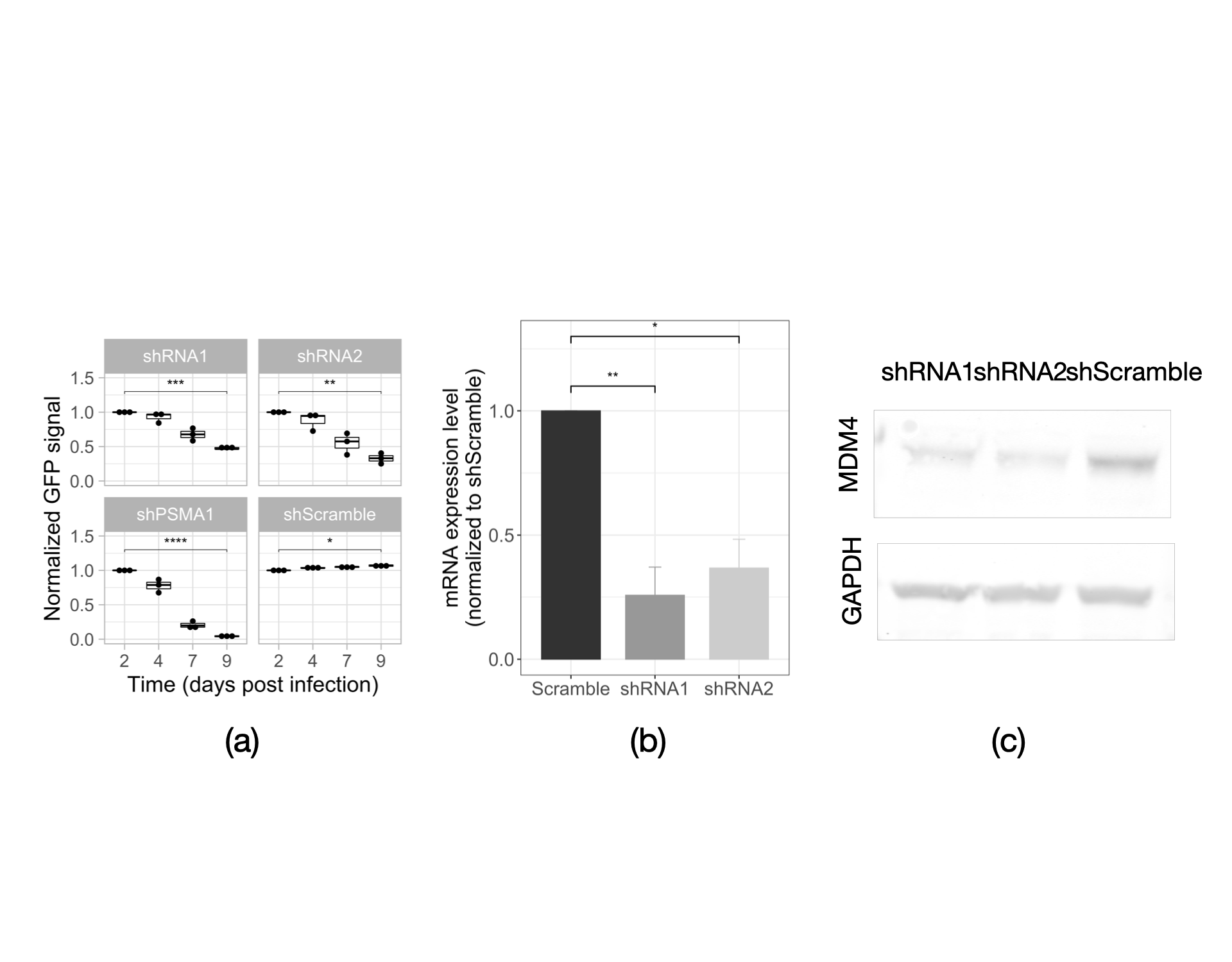

Supplement: Supplementary file 1 [file cells-11-00854-s001.zip › cells-1544439 supplementary new/figureS4_211222.png]

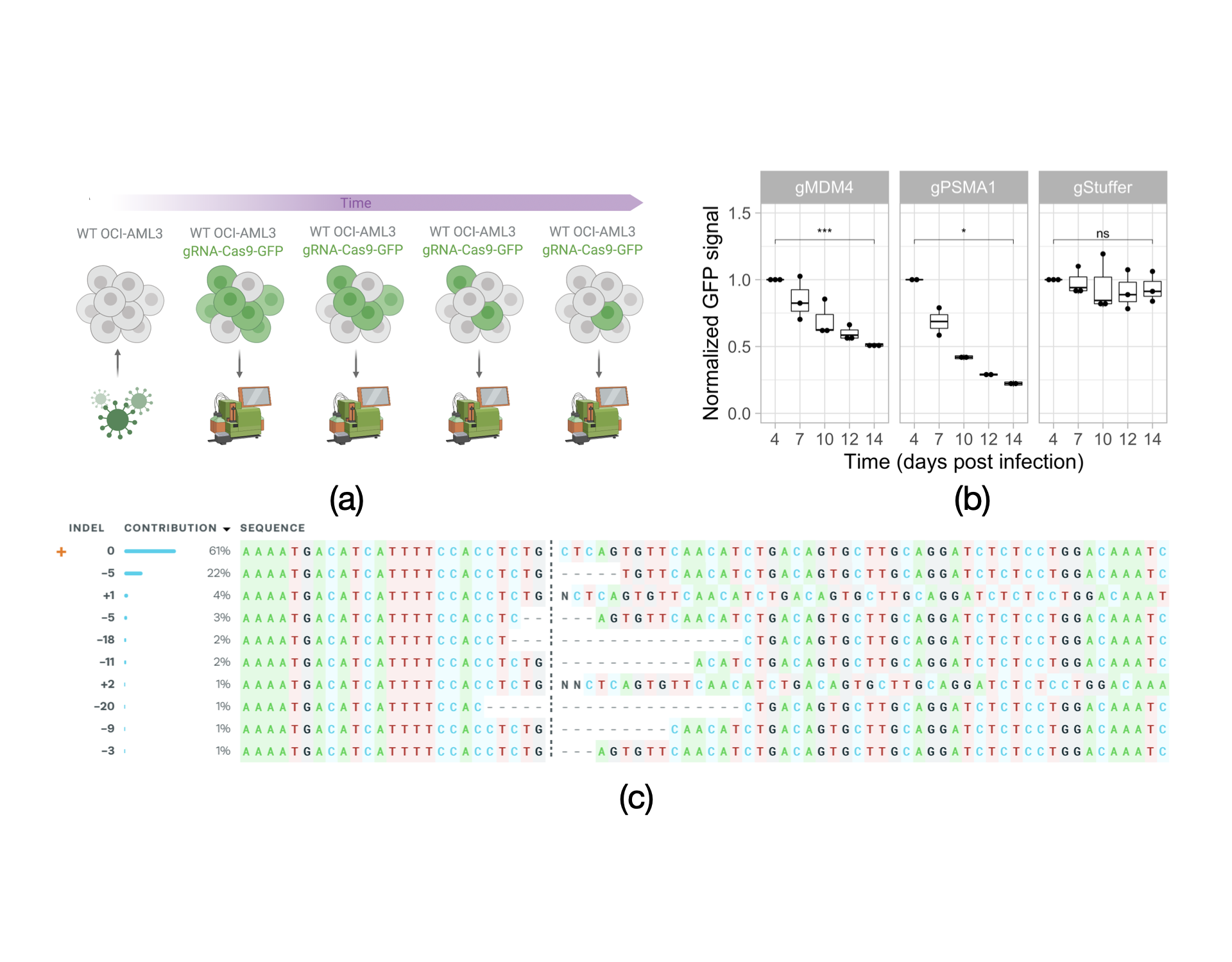

Supplement: Supplementary file 1 [file cells-11-00854-s001.zip › cells-1544439 supplementary new/figureS5_211222.png]

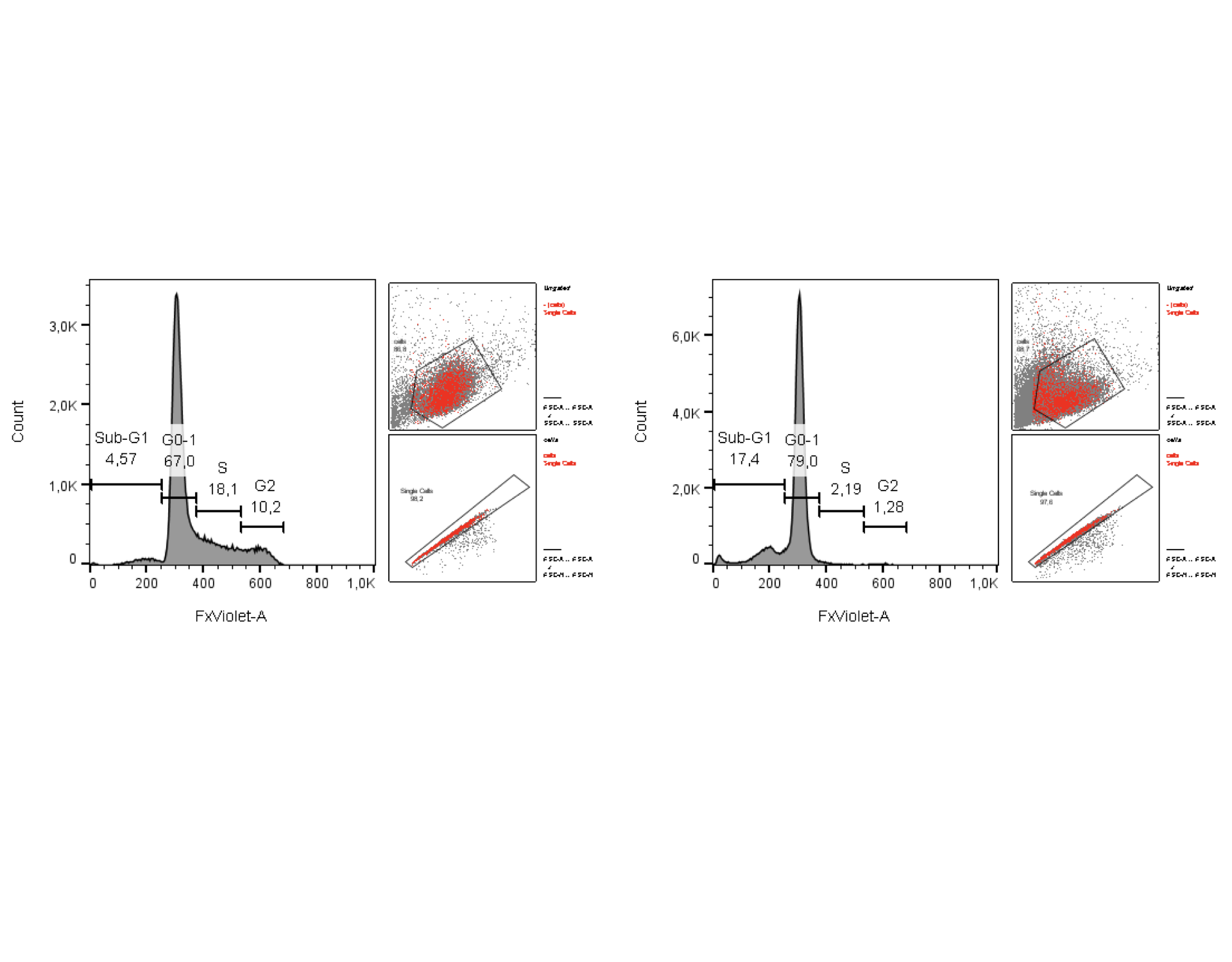

Supplement: Supplementary file 1 [file cells-11-00854-s001.zip › cells-1544439 supplementary new/figureS6.png]
